# Supplementary material for: Earliest amniote tracks recalibrate the timeline of tetrapod evolution
Source: Nature. 2025 May 14;641(8065):1193–200. doi: 10.1038/s41586-025-08884-5 (PMC12119326; doi:10.1038/s41586-025-08884-5)
Supplement: Supplementary file 1 — Supplementary Notes 1–4, Figs. 1 and 2, Table 1 and References. [file 41586_2025_8884_MOESM1_ESM.pdf]

nature portfolio

https://doi.org/10.1038/s41586-025-08884-5

Supplementary information

Earliest amniote tracks recalibrate the timeline of tetrapod evolution

In the format provided by the authors and unedited

Supplementary Information

Part 1: supplementary molecular references

This is a list of the molecular phylogenies relevant to the dating of the amniote crown-group node, tetrapod crown-group node, and lungfish-tetrapod node, curated by www.timetree.org and used in the node dating referenced in the text.

Alfaro, M. E. et al. Nine exceptional radiations plus high turnover explain species diversity in jawed vertebrates. *Proceedings of the National Acadademy of Sciences of the USA* **106**, 13410–13414 (2009).

Amer, S. A. M. & Kumazawa, Y. Timing of a mtDNA gene rearrangement and intercontinental dispersal of varanid lizards. *Genes & Genetic Systems* **83**, 275–280 (2008).

Aris-Brosou, S. & Yang, Z. Effects of models of rate evolution on estimation of divergence dates with special reference to the metazoan 18S ribosomal RNA phylogeny. *Systematic Biology* **51**, 703–714 (2002).

Berbee, M. L. & Taylor, J. W. Dating the molecular clock in fungi – how close are we? *Fungal Biology Reviews* **24**, 1–16 (2010).

Betancur-R, R. et al. The tree of life and a new classification of bony fishes. *PLoS Currents* 5, ecurrents.tol.53ba26640df0ccaee75bb165c8c26288 (2013).

Betancur-R, R., Ortí, G. & Pyron, R. A. Fossil-based comparative analyses reveal ancient marine ancestry erased by extinction in ray-finned fishes. *Ecology Letters* **18**, 441–450 (2015).

Blair, J. E. & Hedges, S. B. Molecular phylogeny and divergence times of deuterostome animals. *Molecular Biology and Evolution* **22**, 2275–2284 (2005).

Chen, M., Zou, M., Yang, L. & He, S. Basal jawed vertebrate phylogenomics using transcriptomic data from Solexa sequencing. *PLoS ONE* **4**, e36256 (2012).

Crottini, A. et al. Vertebrate time-tree elucidates the biogeographic pattern of a major biotic change around the K-T boundary in Madagascar. *Proceedings of the National Acadademy of Sciences of the USA* **109**, 5358–5363 (2012).

Delsuc., F. et al. A phylogenomic framework and timescale for comparative studies of tunicates. *BMC Biology* **16**, 39 (2018).

dos Reis, M. et al. Uncertainty in the Timing of Origin of Animals and the Limits of Precision in Molecular Timescales. *Current Biology* **25**, 2939–2950 (2015).

Feng, Y.-J. et al. Phylogenomics reveals rapid, simultaneous diversification of three major clades of Gondwanan frogs at the Cretaceous-Paleogene boundary. *Proceedings of the National Acadademy of Sciences of the USA* **114**, E5864–E5870 (2017).

Hallström, B. M. & Janke, A. Gnathostome phylogenomics utilizing lungfish EST sequences. *Molecular Biology and Evolution* **26**, 463–471 (2009).

Hedges, S. B. & Kumar, S. (Eds.) *The Timetree of Life*. Oxford University Press (2009).

Hime, P. M. et al. Phylogenomics Reveals Ancient Gene Tree Discordance in the Amphibian Tree of Life. *Systematic Biology* **70**, 49–66 (2021).

Hugall, A. F., Foster, R. & Lee, M. S. Y. Calibration choice, rate smoothing, and the pattern of tetrapod diversification according to the long nuclear gene RAG-1. *Systematic Biology* **56**, 543–563 (2007).

Hughes, L. C. et al. Comprehensive phylogeny of ray-finned fishes (Actinopterygii) based on transcriptomic and genomic data. *Proceedings of the National Acadademy of Sciences of the USA* **115**, 6249–6254 (2018).

Igawa, T., Kurabayashi, A., Usuki, C., Fuji, T. & Sumida, M. Complete mitochondrial genomes of three neobatrachian anurans: a case study of divergence time estimation using different data and calibration settings. *Gene* **407**, 116–119 (2007).

Inoue, J. G. et al. Evolutionary Origin and Phylogeny of the Modern Holocephalans (Chondrichthyes: Chimaeriformes): A Mitogenomic Perspective. *Molecular Biology and Evolution* **27**, 2576–2586 (2010).

Kumar, S. & Hedges, S. B. A molecular timescale for vertebrate evolution. *Nature* **392**, 917–920 (1998).

Kumazawa, Y. Mitochondrial genomes from major lizard families suggest their phylogenetic relationships and ancient radiations. *Gene* **388**, 19–26 (2007).

Laurin, M., Océane, L. & Marjanović, D. What do ossification sequences tell us about the origin of extant amphibians? *Peer Community Journal* **2**, e12 (2022).

Liu, Z. Z. et al. Bioinformatics analysis of melanocortin-1 receptor gene for silver fox and other species. *Journal of Animal and Veterinary Advances* **11**, 1000–1006 (2012).

Mannen, H. & Li, S. S. Molecular evidence for a clade of turtles. *Molecular Phylogenetics and Evolution* **13**, 144–148 (1999).

Meredith, R. W. et al. Impacts of the Cretaceous Terrestrial Revolution and KPg Extinction on Mammal Diversification. *Science* **334**, 521–524 (2011).

Okajima, Y. & Kumazawa, Y. Mitogenomic perspectives into iguanid phylogeny and biogeography: Gondwanan vicariance for the origin of Madagascan oplurines. *Gene* **441**, 28–35 (2009).

Okajima, Y. & Kumazawa, Y. Mitochondrial genomes of acrodont lizards: timing of gene rearrangements and phylogenetic and biogeographic implications. *BMC Evolutionary Biology* **10**, 141 (2010).

Parfrey, L. W., Lahr, D. J. G., Knoll, A. H. & Katz, L. A. Estimating the timing of early eukaryotic diversification with multigene molecular clocks. *Proceedings of the National Acadademy of Sciences of the USA* **108**, 13624–13629 (2011).

Pereira, S. L. & Baker, A. J. A mitogenomic timescale for birds detects variable phylogenetic rates of molecular evolution and refutes the standard molecular clock. *Molecular Biology and Evolution* **23**, 1731–1740 (2006).

Phillips, M. J., Bennett, T. H. & Lee, M. S. Y. Molecules, morphology, and ecology indicate a recent, amphibious ancestry for echidnas. *Proceedings of the National Acadademy of Sciences of the USA* **106**, 17089–17094 (2009).

Pyron, R. A. A likelihood method for assessing molecular divergence time estimates and the placement of fossil calibrations. *Systematic Biology* **59**, 185–194 (2010).

Roelants, K. et al. Identical skin toxins by convergent molecular adaptation in frogs. *Current Biology* **20**, 125–130 (2010).

San Mauro, D. A multilocus timescale for the origin of extant amphibians. *Molecular Phylogenetics and Evolution* **56**, 554–561 (2010).

Shen, X.-X., Liang, D., Wen, J.-Z. & Zhang, P. Multiple genome alignments facilitate development of NPCL markers: a case study of tetrapod phylogeny focusing on the position of turtles. *Molecular Biology and Evolution* **28**, 3237–3252 (2011).

Shen, X.-X., Liang, D. & Zhang, P. The development of three long universal nuclear protein-coding locus markers and their application to osteichthyan phylogenetics with nested PCR. *PLoS ONE* **7**, e39256 (2012).

Subramanian, S., Hunyen, L., Millar, C. D. & Lambert, D. M. Next generation sequencing and analysis of a conserved transcriptome of New Zealand's kiwi. *BMC Evolutionary Biology* **10**, 387 (2010).

Vieites, D. R., Min, M.-S. & Wake, D. B. Rapid diversification and dispersal during periods of global warming by plethodontid salamanders. *Proceedings of the National Acadademy of Sciences of the USA* **104**, 19903–19907 (2007).

Wang, Z. et al. The draft genomes of soft-shell turtle and green sea turtle yield insights into the development and evolution of the turtle-specific body plan. *Nature Genetics* **45**, 701–706 (2013).

Zhang, P. & Wake, D. B. Higher-level salamander relationships and divergence dates inferred from complete mitochondrial genomes. *Molecular Phylogenetics and Evolution* **53**, 492–508 (2009).

Zhang, P., Zhou, H., Chen, Y.-Q., Liu, Y.-F. & Qu, L.-H. Mitogenomic perspectives on the origin and phylogeny of living amphibians. *Systematic Biology* **54**, 391–400 (2005).

Zhang, P., Zhou, H., Liang, D., Liu, Y.-F., Chen, Y.-Q., & Qu, L.-H. The complete mitochondrial genome of a tree frog, *Polypedates megacephalus* (Amphibia: Anura: Rhacophoridae), and a novel gene organization in living amphibians. *Gene* **346**, 133–143 (2005).

Part 2: the geological history of the Lachlan Fold Belt and the age of the Snowy Plains Formation

The footprint slab comes from the Home Station Sandstone Member of the Snowy Plains Formation of the Mansfield Group, which is located in central Victoria within the Lachlan Fold Belt. The locality (see photo below) is in relatively flat, gently undulating, vegetated terrain, with bedrock exposed only along the river banks. The Snowy Plains Formation extends for several kilometres in every direction. The slab itself is essentially pristine and shows no sign of long-distance transport. It is lithologically similar to Home Station Sandstone outcropping in the immediate neighbourhood. We conclude from these observations that the slab can be securely assigned to the Home Station Sandstone. This provides a robust context for determining its age.

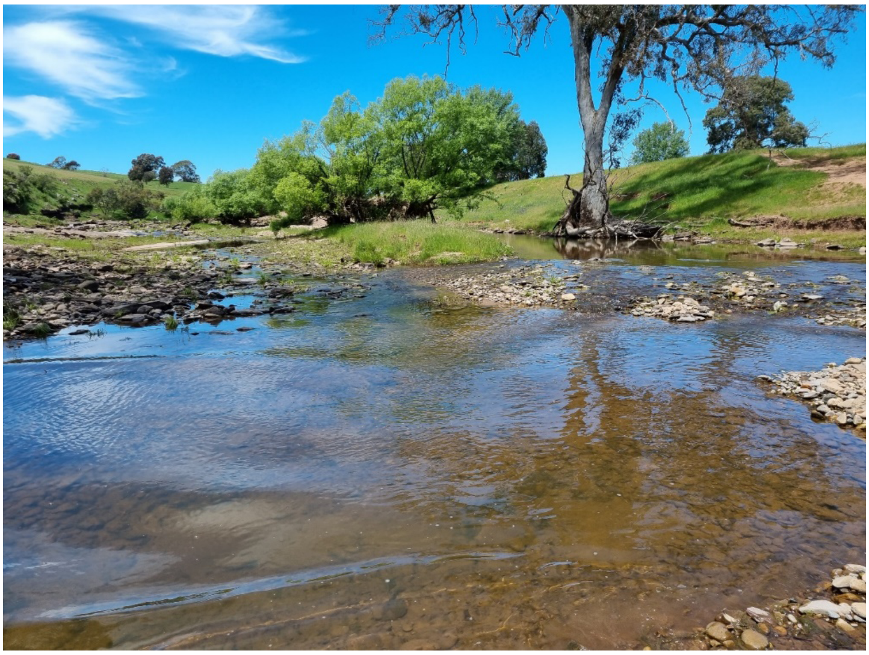

Supp. Info., Fig. 1. The locality on the Broken River where the trackway slab was found. In the Taungurung language this section of the Broken River was known as *Berrepit*. Photo by John Eason.

The Lachlan Fold Belt or Lachlan Orogen is a structurally complex region, extending northwards from Tasmania approximately to the border between New South Wales and Queensland (Foster & Gray 2000). Its Palaeozoic history comprises a series of tectonic events, challenging to categorise as many appear to have been local and/or diachronous, and shifting patterns of sedimentation. Different areas of the Lachlan Fold Belt have distinct sedimentary and tectonic histories, further underscoring the complexity of the region; the area of interest for the present paper is the south-eastern part, comprising southern and central Victoria as well as southernmost New South Wales.

The Mansfield Group sediments were deposited within the Mount Howitt Graben, part of a wider depositional landscape dominated by fluvial deposition that extended northwards past Cobar and eastwards as far as Eden on the New South Wales coast. There was extensive Devonian sedimentation in this region, which contains a number of important fossil localities with characteristic Middle to Late Devonian vertebrate faunas (Long et al. 2021). Among the youngest of these Devonian deposits is the Worange Point Formation of the Merimbula Group, which outcrops on the New South Wales coast near Eden and has recently yielded a

characteristic Famennian vertebrate assemblage including the giant tristichopterid lobe-fin *Edenopteron* and the antiarch *Remigolepis* (Young et al. 2013).

The base of the Mansfield Group may be Devonian in age (Marsden 1988), but the upper part of the group contains a vertebrate assemblage that lacks diagnostic Devonian groups such as tristichopterids and antiarchs, and should thus post-date the end-Devonian extinction event. Members of the assemblage include the large acanthodian *Gyracanthides* and two smaller forms, the rhizodont lobe-fin *Barameda*, the lungfish *Delatitia*, the actinopterygians *Mansfieldiscus* and *Novogonatodus*, and several chondrichthyans including the distinctive tooth genus *Ageleodus* (Garvey & Turner 2006). The overall character of this assemblage is unmistakably Early Carboniferous. Its maximum age is thus set by the Devonian-Carboniferous (Famennian-Tournaisian) boundary, currently dated to 358.9 million years.

The minimum age for the Mansfield Group, which is of critical importance for the argumentation presented in this paper, is less precisely constrained. The lack of any obvious hiatus in sedimentation across the Devonian-Carboniferous boundary argues for an earliest Carboniferous age. The single known *Ageleodus* tooth most closely resembles the *Ageleodus* material from the Famennian Catskill Formation of Pennsylvania, rather than later Carboniferous species of this long-lived genus (Garvey & Turner 2006). However, the most robust minimum age constraint is imposed by the Kanimblan Orogeny, which affected the entire region. The Mansfield Group certainly predates the main phase of this orogeny. It has not been dated in the immediate vicinity of the Mansfield Group outcrops, but good data are available from the Hill End Trough and adjacent Capertree High some 500 km to the north-east (Fergusson & Colquhoun 2021). There, Kanimblan deformation seems to have begun in the latest Devonian and continued into the Tournaisian; the intrusion of a series of Bathurst-type granites with U-Pb zircon dates of 341-314 million years, i.e. late Tournaisian to early Moscovian (and with a single, early Tournaisian, outlier of 358 million years) mark the end of the Orogeny (Fergusson & Colquhoun 2021). If this timeframe also applies to the area of the Mansfield Group, which seems likely as the Kanimblan Orogeny appears to have been a widespread and brief event (Foster & Gray 2000), and we allow for continued deposition only during the earliest stage of deformation, the Snowy Plains Formation cannot be later than early Tournaisian. An age similar to the earliest crown-group amniote tracks from Euramerica, in other words Serpukhovian to Bashkirian (Górecka-Nowak & Majewska 2002; Marchetti et al. 2020), is conclusively ruled out.

References

Fergusson, C. L. & Colquhoun, G. P. 2021. Devonian–Carboniferous regional deformation in the northeastern Lachlan Orogen, southeastern Australia. *Australian Journal of Earth Sciences* **68**, 1092–1110.

Foster, D. A. & Gray, D. R. 2000. Evolution and structure of the Lachlan Fold Belt (Orogen) of Eastern Australia. *Annual Review of Earth and Planetary Sciences* **28**, 47–80.

Garvey, J. M. & Turner, S. 2006. Vertebrate microremains from the presumed earliest Carboniferous of the Mansfield Basin, Victoria. *Alcheringa* **30**, 43–62.

Górecka-Nowak, A. & Majewska, M. 2002. Remarks on palynostratigraphy of the Namurian Wałbrzych Formation in the northern part of the Intrasudetic Basin (SW Poland). *Geological Quarterly* **46**, 101–115.

Long, J. A., Thomson, V., Burrow, C. J. & Turner, S. 2021. Fossil chondrichthyan remains from the Middle Devonian Kevington Creek Formation, South Blue Range, Victoria. *In* Pradel, A., Denton, S. S. & Janvier, P (eds.) *Ancient Fishes and their Living Relatives: a Tribute to John G. Maisey,* 239–245. Verlag Dr. Friedrich Pfeil, München, Germany.

Marchetti, L., Voigt, S., Lucas, S. G., Stimson, M. R., King, O. A. & Calder, J. H. 2020. Footprints of the earliest reptiles: *Notalacerta missouriensis* – ichnotaxonomy, potential trackmakers, biostratigraphy, palaeobiogeography and palaeoecology. *Annales Societatis Geologorum Poloniae* **90**, 271–290.

Marsden, M. A. H. 1988. Upper Devonian-Carboniferous. *In* Douglas, J. G. & Ferguson, J. A. (eds.) *Geology of Victoria*, 147–194. Victorian Division of the Geological Society of Australia Incorporated, Melbourne, Australia.

Young, B., Dunstone, R. L., Senden, T. J. & Young, G. C. 2013. A gigantic sarcopterygian (tetrapodomorph lobe-finned fish) from the Upper Devonian of Gondwana (Eden, New South Wales, Australia). *PLoS ONE* **8**, e53871.

Part 3: track measurements and comparisons with known sauropsid ichnotaxa

Trackway A	Footprint L	Footprint W	Divarication angle between digits I and V	Pace angulation	Pace length	Trackway width
Ap1	-	-	-	-	-	-
Am1	40	54	95°	-	-	-
Ap2	45	53	120°	75°	-	85
Am2	37	41	-	92°	-	74
Ap3	38	44	-	89°	156	90
Am3	44	51	120°	112°	164	73
Ap4	46	53	100°	-	159	-
Am4	40	51	-	-	171	-
Average	41	50	109°	92°	163	81
Trackway B						
Bm1	-	-	-	-	-	-
Bp1	-	-	-	-	-	-
Bm2	-	-	-	78°	-	80
Bp2	-	-	-	75°	-	75
Bm3	-	-	-	76°	112	79
Bp3	-	-	-	78°	108	73
Bm4	-	-	-	82°	134	59
Bp4	-	-	-	-	105	-
Bm5	-	-	-	-	123	-
Average				78°	116	73
Isolated print	45	64	125°	-	-	-
Dromopus lacertoides NSM 009 GF 012	37	27.3	164°	99.57°	189.5	
YPM 519	59.9	43.8	140.5°	107.9°	315.3	
Varanopus microdactylus NSM 997 GF30.6	19.2	24.5	120.2°	84°	106.7	
NSM 99 GF 34b	28.1	28.7	98.5°	90.3°	130.8	
Varanopus diagnosis (Voigt , 2005)	<45mm		80-90 on pes, 120 on hand	75-100° for the pes, 80-115° for the hand		

Supp. Info., Table 1. Measurements for the Snowy Plains Formation amniote trackways, all linear measurements in mm, angular measures in degrees. NSM= Nova Scotia Museum, Halifax, Canada and YPM= Yale Peabody Museum, New Haven, Connecticut, United States. Measurements for D. lacertoides and V. microdactylus are taken from Marchetti et al. (2021). Measurement conventions follow Casamiquela et al. (1987) where possible.

Comparison to existing ichnogenera

The Snowy Plains Formation (SPF) tracks match closely with Haubold and Lucas' (2003) diagnosis for *Varanopus*, to the exclusion of *Erpetopus*, in that pes digit V is as long as pes digit III and does not display outward curvature but the SPF tracks are almost twice as large (avg. manus and pes 41mm in L and 50 mm in width) as those of *Varanopus* (L- 20 mm and W- 25mm) and have a wider divarication angle between digits I and V (avg. 109° in the former and 90° in the latter). However, in the *Varanopus curvidactylus* holotype (YPM 1106) figured by Haubold and Lucas (2003, Fig. 4.2) the divarication angle appears to be ~120° and in an additional example (Fig. 4.3) it appears to be 105°. It thus appears that the morphology of the SPF tracks fit well within the range seen in *Varanopus* and differ only in size. The protocols for measuring digit divarication are also notoriously problematic, with few authors employing a replicable methodology (Camens and Worthy, 2019) and so diagnoses based on divarication angles should be interpreted with care.

Voigt (2005) further summarised the diagnostic characters for *Varanopus,* including indentations up to 45mm in length, which are of comparable size to the SPF tracks.

References

Camens, A. B. & Worthy, T. H. 2019. Pliocene Avian Footprints from the Lake Eyre Basin,

South Australia. *Journal of Vertebrate Paleontology,* **39,** e1676764.

Casamiquela, R. M., Demathieu, G.R., Haubold, H., Leonardi, G. & Sarjeant, W. A. S. 1987. *Glossary and manual of tetrapod footprint palaeoichnology,* Ed. Leonardi, G., Brazil, Conselho Nacional de Desenvolvimento Cientifico e Teconologico.

Haubold, H. 1996. Ichnotaxonomie und Klassifikation von Tetrapodenfährten aus dem Perm. *Hallesches Jahrbuch für Geowissenschaften,* **18,** 22–88.

Haubold, H. & Lucas, S. G. 2003. Tetrapod footprints of the Lower Permian Choza Formation at Castle Peak, Texas. *Paläontologische Zeitschrift,* **77,** 247–261.

Marchetti, L., Voigt, S., Buchwitz, M., Macdougall, M. J., Lucas, S. G., Fillmore, D. L., Stimson, M. R., King, O. A., Calder, J. H. & Fröbisch, J. 2021. Tracking the origin and early evolution of reptiles. *Frontiers in Ecology and Evolution,* **9,** 696511, 1–31.

Voigt, S. 2005. *Die tetrapodenichnofauna des kontinentalen oberkarbon und perm im thüringer Wald-ichnotaxonomie, paläoökologie und biostratigraphie*, Cuvillier Verlag.

Notes relating to ichnotaxonomic identification:

Diagnostic characters of *Varanopus* (cited from Haubold & Lucas 2003)

(Note that the definition of "Choza Formation" used by Haubold & Lucas 2003 is disputed, and that the deposit yielding the tracks is better designated as the upper Clear Fork Formation (uppermost Cisuralian/Leonardian). See the following references:

Hentz, T. F. 1988. Lithostratigraphy and paleoenvironments of Upper Paleozoic continental red beds, north central Texas: Bowie (new) and Wichita (revised) groups. *Bureau of Economic Geology: Report of Investigations*, **170**, 55 pp.

Nelson, W.J., R.W. Hook, and D.S. Chaney. 2013. Lithostratigraphy of the Lower Permian (Leonardian) Clear Fork Formation of north-central Texas. *New Mexico Museum of Natural History and Science, Bulletin* **60**, 286–311.)

"Characteristics: The significant characters of *Varanopus* became understandable from a specimen collected in 1999 (NMMNH P-32390-392: Figs. 4.3, 6C). Pes and manus measure about 20 mm in length and 25 mm in width, parallel and perpendicular to extension of digit III. Overall pattern of manus and pes, and trackway as well, resembles *Erpetopus.* However, pes digit V is as long as digit III. Angle between axis of digit I and V is 90 °. Digit V is well integrated into pes, which is thus structured by a close group of all five digits. Manus shows same arrangement, although digit V is as short as II, but very close to digit IV. Recorded integration of digit V of both manus and pes, with evidence of origin of all digits from sole and plantar impression, shows that length of digit V is anatomically controlled, and not due to deformation or displacement by extramorphology or gait.

Discussion: In addition to the *Erpetopus* tracks at Castle Peak, there are some tracks that show only a few different proportions of the digits--pes digit V is longer. The remaining morphology is similar. In the Castle Peak assemblage, these seem to be only larger tracks of *Erpetopus.* Moodie separated these forms from *Erpetopus* and *Microsauropus* as *Varanopus curvidactylus.* But, Sarjeant (1971) diagnosed *Varanopus* erroneously and illustrated as *V. curvidactylus* specimens that belong to *Erpetopus* and *Microsauropus.* This mistake becomes clear after examination of the holotype of *V. curvidactylus* (YPM 1106: Figs. 4.2, 6B). Moodie (1929: fig. 8) illustrated from YPM 1106 only the sketch of a manus-pes set. In the description, he mentioned the pes with a 20 mm length, and the specimen displays a relatively long digit V, which is placed more outward but with the same principal direction as digits I to IV. The outward curvature of digit V noted in the diagnosis of Sarjeant (1971) is not present. The outward curvature of pes digit V is instead a character of *Erpetopus* (= *Microsauropus),* so it is important for the differentiation of *Erpetopus* from *Varanopus.*

The taxonomic separation of *Varanopus* from *Erpetopus* is based on the proportions of the digits, and the position of the digits, in particular pes digit V. The minor difference of size is diagnostically irrelevant. Only four fragmentary specimens were available to Moodie because tracks of *V. curvidactylus* are rare at Castle Peak. However, the few specimens show a distinct and clear record of all details. This is due to the size of the tracks, which are above the critical length of 20 mm.

Compared to *Erpetopus,* these tracks were less influenced by extramorphological factors. If the study is restricted to specimens from the Choza Formation, a separate ichnospecies *curvidactylus* within the genus *Erpetopus* might represent the difference sufficiently. But, with regard to comparable forms from other Permian formations, the

separation of the ichnogenera by Moodie appears useful and should be retained. The proportions of the digits and the resulting morphology of the manus and pes imprints are of ichnogeneric value in Permian tetrapod tracks (Haubold 1996).

Comparison of *Erpetopus* and *Varanopus* to osteologically known forms from the Early Permian points to small reptiles such as protorothyridids and captorhinids as the trackmakers. Captorhinids are known from skeletal fossils from the Choza Formation (Olson 1958; Murry & Johnson 1987), but no more detailed relationship to any skeletally known form is possible. Indeed, *Erpetopus* and *Varanopus* show a well-established but relatively conservative arrangement of pentadactyl autopods and regular trackway pattern that is common in several early amniotes."

Diagnostic characters of *Varanopus*, cited from Voigt (2005) with English translation

"Charakteristik (nach Haubold 1971a; Haubold & Lucas 2001b, 2003; emend.; Abb. 37-39): Quadrupede Fährten mit pentadactylen, semiplantigraden Eindrücken bis ca. 45 mm Fußlänge. Fuß rund ein Fünftel länger als die Hand. Zehen gerade bis leicht einwärts gekrümmt mit spitz zulaufendem Klauenabdruck, der stumpfwinklig um 140° zum Zehenstrahl nach innen orientiert ist. Häufiger extramorphologisch bedingte Bifurkation der Zehenenden. Klauenabdruck des fünften Zehs parallel zur Zehenachse, selten auswärts gerichtet. Zehenlänge an Hand und Fuß von I nach IV zunehmend, Zeh V am Fuß etwa so lang wie III, an der Hand zwischen I und II. Länge-Breite-Verhältnis für den Fuß rund 1 : 1, Hand geringfügig breiter als lang, ca. 0,9 : 1. Zehenwinkel I-V am Fuß zwischen 80 und 90°, an der Hand um 120°. Sohle auffallend kurz, macht etwa ein Fünftel der Gesamtlänge des Eindruckes aus. Distale Begrenzung der Eindrücke gerade oder konkav. Aus Innenbelastung resultiert breite Ausbildung der Zehen I bis III. Zeh V am Fuß häufig schmal und ohne Kontakt zur Sohle. Soweit bekannt, zeigen die Fährten ausschließlich alternierende Anordnung von Hand-Fuß-Eindruckpaaren. Primäres Übertreten ist möglich. Relation D : A gleich 0,6-1,4 : 1, im Mittel 0,84 : 1. Fuß parallel zur Mittellinie, Hand durchschnittlich 10° einwärts orientiert. Pace und Schrittbreite des Fußes etwas größer als die der Hand. Schrittwinkel 75-100° für den pes, 80-115° für die Hand. Dokumentierte Rumpflängen 75-100 mm. Stride zu Rumpflänge gleich 1,2-1,4 : 1, Stride zu Fußlänge 3,6-4,7 : 1. Accessoires wie Hautabdrücke oder Schwanzschleifspuren sind nicht belegt.

Characteristics: (according to Haubold 1971a; Haubold & Lucas 2001b, 2003; emend.; Fig. 37-39): Quadrupedal tracks with pentadactyl, semi-plantigrade footprints up to approx. 45 mm foot length. Pes about a fifth longer than the manus. Toes straight to slightly curved with a pointed claw print that is oriented inwards at an obtuse angle of 140° to the toe long axis. Extramorphologically caused bifurcations of toe ends frequently seen. Claw impression of the fifth toe parallel to the axis of the toe, rarely directed oputwards. Toe length on manus and pes increasing from I to IV, toe V on pes about the same length as digit III, on the hand between I and II. Length-width ratio for the pes around 1: 1, manus slightly wider than long, approx. 0.9: 1. Toe angle I-V on foot between 80 and 90°, on the hand approximately 120°. Sole is noticeably short, making up about a fifth of the total length of the impression. Distal boundary of the impressions straight or concave. Pressure on the toes results in wide impressions of toes I to III. Toe V on the foot often narrow and without contact with the sole. As far as is known, the tracks only show alternating arrangements of manus-pes impression pairs. Primary overstepping is possible. Relation D: A equal to 0.6-1.4: 1, median value 0.84: 1. Pes parallel to the center line, manus rotated inward by on average 10°. Pace and stride width of the pes slightly greater than that of the manus. Step angle 75-100° for the pes, 80-115° for the manus. Documented torso lengths 75-100 mm. Stride to torso length equal to l.2-1.4: 1, Stride to foot length 3.6-4.7: 1. Accessories such as skin impressions or tail drag marks are not documented."

Part 4: data about the Early Carboniferous tetrapod tracks from the Intra-Sudetic Basin Silesia, Poland

The volcano-sedimentary successions of the Intra-Sudetic Basin (Czech Republic and Poland) and the adjacent Karkonoše-Piedmont Basin (Czech Republic) preserve one of the best Late Carboniferous to Early Permian tetrapod trackway fossil records in the world. These areas are historically important for the study of Late Palaeozoic tetrapod tracks (Geinitz, 1861, 1863; Geinitz and Deichmüller, 1882; Fritsch, 1895, 1901; Czyżewska, 1955; Holub and Kozur, 1981; Ptaszyński and Niedźwiedzki, 2004; Voigt et al., 2012, 2024; Niedźwiedzki and Bojanowski, 2012; Sadlok, 2023; Niedźwiedzki et al., on-going study). This region's sedimentary rocks were deposited in a wide diversity of palaeoecological conditions, preserve abundant plant remains, invertebrate, fish and tetrapod remains as well paleosol horizons that can be correlated with tetrapod trace fossils (e.g. Zajíc, 2014; Opluštil et al., 2016, 2022).

Despite the long history of collection in the Intra-Sudetic Basin, many fossil specimens (e.g. tetrapod bones, tracks and coprolites) and many sites remain undescribed. This includes trace fossil specimens collected from the Wałbrzych Formation, which represents Early Carboniferous (mid-Serpukhovian) to Late Carboniferous (early Baskhirian) coal-bearing interval within the Lower Carboniferous to Lower Permian volcano-sedimentary succession of the Intra-Sudetic Basin. Many geological studies on the Wałbrzych Formations were published during the last 100 year and our understanding of the age and sedimentary environment of this unite is sufficient (e.g., Grocholski, 1960, 1963, 1974; Dziedzic, 1971; Nemec; 1984; Mastalerz K., 1987; Mastalerz M., 1992; Bossowski et al., 1995; Mastalerz and Prouza, 1995; Nowak, 2000; Górecka-Nowak and Majewska, 2002, 2003; Uglik and Nowak, 2015; Górecka-Nowak et al., 2021).

Recent palynostratigraphic studies of the Wałbrzych Formation (Górecka-Nowak et al. 2021) have provided new data on their age, which revises previous dating (see Górecka 1962, 1968; Górecka-Nowak and Majewska, 2002, 2003). The studied surface exposures of the Wałbrzych Formation were included in three Middle-Upper Namurian A miospore subzones, which are correlated with Middle to Late Serpukhovian and the Early Bashkirian. The boundary between the Mississippian and Pennsylvanian, correlated to the Serpukhovian/Bashkirian boundary is probably located in the upper part of the Wałbrzych Formation (Górecka-Nowak et al., 2021).

The tetrapod tracks illustrated in this study (Fig. 3a-c in main text) come from outcrops and post mining areas (Konradów and Szczawno Zdrój old quarries and coal mine dumps) of the lower and middle part of the Wałbrzych Formation (Supp. Info., Fig. 2), their stratigraphic position, which is based on miospore studies, indicates the Middle to Late Serpukhovian age (see Górecka-Nowak et al., 2021: Konradów and Szczawno-Zdrój sections).

A full, comprehensive study of tetrapod bones, tracks, coprolites collected from the Wałbrzych Formation, in total 5 sites, 39 specimens, is under preparation (Niedźwiedzki et al., on-going study). This material represents the oldest record of tetrapods from the Upper Palaeozoic sedimentary succession of the Intra-Sudetic Basin and in more general from the Sudetes region. The origin of amniotes has been placed in the transition from Early to Late Carboniferous (late Mississippian-early Pennsylvanian), (see Pardo et al., 2020; Marchetti et al., 2021). Sudetes material is important for understanding the Carboniferous stage in the evolution of tetrapods and will allow for new interpretations regarding the time of origin of the Early to Late Carboniferous ecosystems with diversified sauropsids and synapsids.

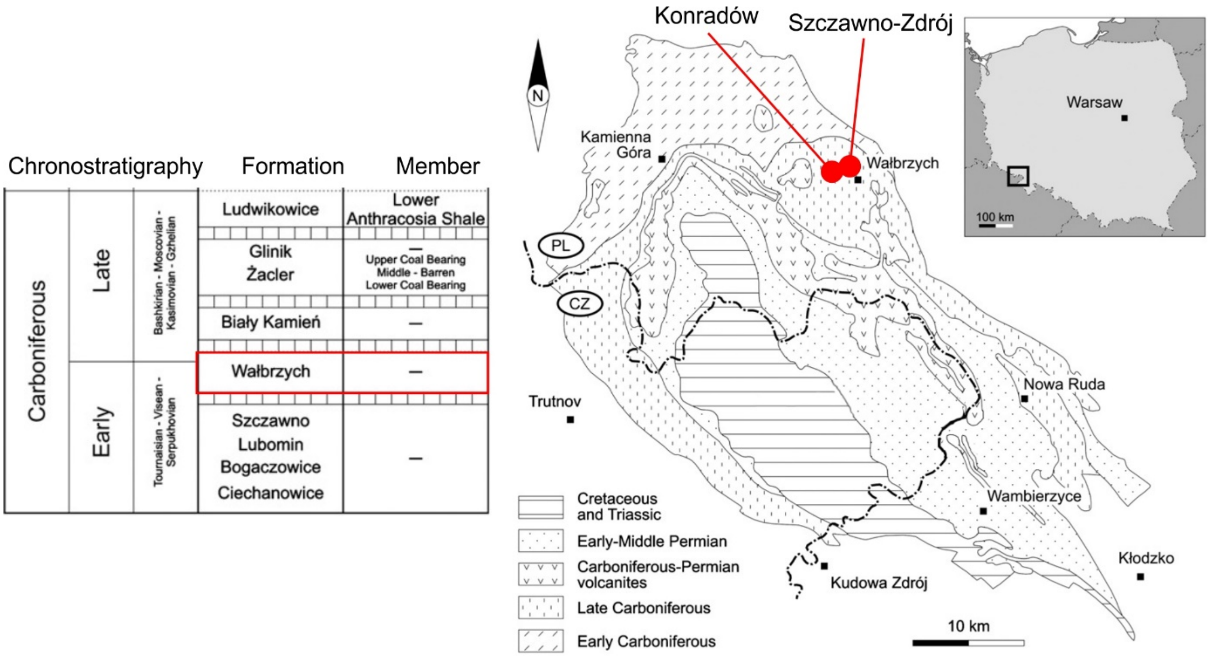

Supp. Info., Fig. 2. Carboniferous lithostratigraphy (Wałbrzych Fm is marked by red frames) in the Polish part of the Intra-Sudetic Basin (after Nemec, 1984; Górecka-Nowak et al., 2021) and simplified geological map of the basin (after Awdankiewicz et al., 2003) and (after Nemec et al. 1982 and Pešek, 2004) showing the position of the Konradów and Szczawno-Zdrój tracksites. Vertical distances in the lithostratigraphic scheme not to scale.

References

Awdankiewicz, M., Kurowski, L., Mastalerz, K., Raczyński, P., 2003. The Intra-Sudetic Basin—a record of sedimentary and volcanic processes in late- to post-orogenic tectonic setting. Geolines 16, 165–183.

Bossowski, A., Ihnatowicz, A., Mastalerz, K., Kurowski, L., Nowak, G.J., 1995. Intra-Sudetic Depression. Prace Państwowego Instytutu Geologicznego, Warszawa, 148, 142–147.

Calábková G, Madzia D, Nosek V, Ivanov M., 2023. Tracking 'transitional' diadectomorphs in the earliest Permian of equatorial Pangea. PeerJ 11, e16603.

Czyżewska, T., 1955. Reptile trackways from the Permian of Wambierzyce in Lower Silesia (Poland). Acta Geologica Polonica 5, 131–160 [in Polish].

Dziedzic, K., 1971. Sedimentation and palaeogeography of the Upper Carboniferous deposits in the Intra-Sudetic Depression. Geologia Sudetica 5, 7–66 [in Polish with English summary].

Fritsch, A., 1895. Über neue Wirbeltiere aus der Permformation Böhmens nebst einer Übersicht der aus derselben bekannt gewordenen Arten. Sitzungsberichte der Königlich Böhmischen Gesellschaft der Wissenschaften, Mathematisch-Naturwissenschaftliche Klasse, 52, pp. 1–17.

Fritsch, A., 1901. Fauna der Gaskohle und der Kalksteine der Permformation Böhmens, IV. Selbstverlag, Prag. 98 pp.

Geinitz, H.B., 1861. Dyas oder die Zechsteinformation und das Rothliegende (Permische Formation zum Theil). W. Engelmann, Leipzig. 130 pp.

Geinitz, H.B., 1863. Beiträge zur Kenntnis der organischen Überreste in der Dyas (oder permischen Formation zum Theil) und über den Namen Dyas. Neues Jahrbuch für Mineralogie, Geologie und Paläontologie 1863, 385–398.

Geinitz, H.B., Deichmüller, J.V., 1882. Die Saurier der unteren Dyas von Sachsen. Palaeontographica 29, 1–46.

Górecka, T., 1962. Niektóre wyniki badań sporowych w wałbrzyskiej niecce węglowej. Kwartalnik Geologiczny 6(4), 785–786 [in Polish].

Górecka, T., 1968. Namurian–Westphalian boundary in the north-west ern part of the Intra-Sudetic trough. Kwartalnik Geologiczny 12(1), 51–64 [in Polish with English summary].

Górecka-Nowak, A., Majewska, M., 2002. Remarks on palynostratigraphy of the Namurian Wałbrzych Formation in the northern part of the Intra-Sudetic Basin (SW Poland). Geological Quarterly 46(2), 101–115.

Górecka-Nowak, A., Majewska, M., 2003. The palynostratigraphy and palynofacies of the Namurian Wałbrzych Formation in the northern part of the Intra-Sudetic Basin (SW Poland). In: Proceedings of the XVth International Congress on Carboniferous and Permian Stratigraphy: 333–342.

Górecka-Nowak, A., Jankowska, A., Muszer, J., 2021. Age revision of Carboniferous rocks in the northern part of the Intra-Sudetic Basin (SW Poland) based on miospore data. Geological Quarterly 65, 8.

Grocholski, A., 1960. Notes on geological structure of western region of Wałbrzych. Kwartalnik Geologiczny 4(3), 631–646 [in Polish with English summary].

Grocholski, A., 1963. Results of geological investigations in the Lower Silesian Coal Basin. Przegląd Geologiczny 11, 323–401 [in Polish with English summary].

Grocholski, A., 1974. Stratigraphical problems of the Silesian in the Lower Silesian Coal Basin. Kwartalnik Geologiczny 18(1), 63–79 [in Polish with English summary].

Holub, V., Kozur, H., 1981. Revision einiger Tetrapodenfährten des Rotliegenden und biostratigraphische Auswertung der Tetrapodenfährten des obersten Karbon und Perm. Geologisch-Paläontologische Mitteilungen Innsbruck 11, 149–193.

Marchetti, L., Voigt, S., Buchwitz, M., MacDougall, M., Lucas, S. G., Fillmore, D. L., Stimson, M., King, O., Calder, J. H., Fröbisch, J., 2021. Tracking the Origin and Early Evolution of Reptiles. Frontiers in Ecology and Evolution 9, 696511.

Mastalerz, K., 1987. Sedimentation in the Intra-Sudetic Basin at the Lower and Upper Carboniferous transition. In: Guide book of the 58-th Meeting of Polish Geological Society at Wałbrzych (eds. Z. Baranowski, A. Grocholski, J. Malinowski, J. Oberc and S. Porębski): 134–145. Zakład Graficzny, Kraków.

Mastalerz, M., 1992. Petrography and depositional conditions of the coal seams of the Wałbrzych Formation, Intra-Sudetic Basin, SW Poland. Geologia Sudetica 26, 47–82.

Nemec, W., 1984. Wałbrzych Beds (Lower Namurian, Wałbrzych coal measures): analysis of alluvial sedimentation in a coal basin. Geologia Sudetica 19, 7–67 [in Polish with English summary].

Nemec, W., Porębski, S.J., Teisseyre, A.K., 1982. Explanatory notes to the lithotectonic molasse profile of the Intra-Sudetic Basin, Polish part. Veröffentlichungen des Zentralinstitutes für Physik der Erde, Akademie der Wissenschaften der DDR 66, 267–278.

Niedźwiedzki, G., et al. A latest Mississippian (late Early Carboniferous) tetrapod fauna from the Intra-Sudetic Basin, Poland (on-going study).

Niedźwiedzki G., Bojanowski M., 2012. A supposed eupelycosaur body impression from the Early Permian of the Intra-Sudetic Basin, Poland. Ichnos 19(3), 150–155.

Nowak, G.J., 2000. Thermal maturity of coals from the Lower Silesian Coal Basin on the background of their petrography and genesis. Biuletyn Państwowego Instytutu Geologicznego 391, 89–146.

Opluštil, S., Šimůnek, Z., Mencl, V. 2022. Macroflora of the Krkonoše-Piedmont Basin (Pennsylvanian–early Permian); Bohemian Massif, Czech Republic. Review of Palaeobotany and Palynology 303, 104665.

Opluštil, S., Schmitz, M., Kachlík, V., Štamberg S., 016. Re-assessment of lithostratigraphy, biostratigraphy and volcanic history of the Late Paleozoic Intra-Sudetic, Krkonoše-Piedmont and Mnichovo Hradiště basins (Czech Republic) based on new U–Pb CA-ID-TIMS ages. Bulletin of Geosciences 91, 399–432.

Pardo, J. D., Lennie, K., Anderson, J. S. 2020. Can we reliably calibrate deep nodes in the tetrapod tree? Case studies in deep tetrapod divergences. Frontiers in Genetics 11, 1159.

Pešek, J., 2004. Late Palaeozoic limnic basins and coal deposits of the Czech Republic. Folia Musei Rerum Naturalium Bohemiae Occidentalis Geologica 1, 1–188.

Ptaszyński, T., Niedźwiedzki, G., 2004. New finds of vertebrate footprints from the Lower Permian of Wambierzyce, Poland. Geological Quarterly 48, 199–202.

Sadlok, G., 2023. Taphonomy of tiny tetrapod tracks in an example from the Lower Permian (Cisuralian) Słupiec Formation (SW Poland). Palaios 38(9), 395–406.

Uglik, M., Nowak, G.J., 2015. Petrological recognition of bituminous inertinite enriched coals of the Lower Silesian Coal Basin (Central Sudetes, SW Poland). International Journal of Coal Geology 139, 49–62.

Voigt, S., Niedźwiedzki, G., Raczyński, P., Mastalerz, K., and Ptaszyński, T. 2012. Early Permian tetrapod ichnofauna from the Intra-Sudetic Basin, SW Poland. Palaeogeography, Palaeoclimatology, Palaeoecology 313–314, 173–180.

Voigt, S., Calábková, G., Ploch, I., Nosek, V., Pawlak, W., Raczyński, P., Spindler, F., Werneburg, R., 2024. A diadectid skin impression and its implications for the evolutionary origin of epidermal scales. Biology Letters 20, 20240041.

Zajíc, J., 2014. Permian fauna of the Krkonoše Piedmont Basin (Bohemian Massif, Central Europe). Acta Mus. Nat. Prague, Series B, Historia Naturalis 70(3-4), 131–142.
