# Supplementary material for: Earliest amniote tracks recalibrate the timeline of tetrapod evolution
Source: Nature. 2025 May 14;641(8065):1193–200. doi: 10.1038/s41586-025-08884-5 (PMC12119326; doi:10.1038/s41586-025-08884-5)
Supplement: Supplementary file 2 — Reporting Summary [file 41586_2025_8884_MOESM2_ESM.pdf]

nature portfolio

Corresponding author(s): Per E. Ahlberg

Last updated by author(s): May 22, 2024

Reporting Summary

Nature Portfolio wishes to improve the reproducibility of the work that we publish. This form provides structure for consistency and transparency in reporting. For further information on Nature Portfolio policies, see our Editorial Policies and the Editorial Policy Checklist.

Statistics

For all statistical analyses, confirm that the following items are present in the figure legend, table legend, main text, or Methods section.

n/a	Confirmed	
☒	☐	The exact sample size (*n*) for each experimental group/condition, given as a discrete number and unit of measurement
☒	☐	A statement on whether measurements were taken from distinct samples or whether the same sample was measured repeatedly
☒	☐	The statistical test(s) used AND whether they are one- or two-sided<br>*Only common tests should be described solely by name; describe more complex techniques in the Methods section.*
☒	☐	A description of all covariates tested
☒	☐	A description of any assumptions or corrections, such as tests of normality and adjustment for multiple comparisons
☒	☐	A full description of the statistical parameters including central tendency (e.g. means) or other basic estimates (e.g. regression coefficient) AND variation (e.g. standard deviation) or associated estimates of uncertainty (e.g. confidence intervals)
☒	☐	For null hypothesis testing, the test statistic (e.g. *F*, *t*, *r*) with confidence intervals, effect sizes, degrees of freedom and *P* value noted<br>*Give P values as exact values whenever suitable.*
☒	☐	For Bayesian analysis, information on the choice of priors and Markov chain Monte Carlo settings
☒	☐	For hierarchical and complex designs, identification of the appropriate level for tests and full reporting of outcomes
☒	☐	Estimates of effect sizes (e.g. Cohen's *d*, Pearson's *r*), indicating how they were calculated

Our web collection on statistics for biologists contains articles on many of the points above.

Software and code

Policy information about availability of computer code

Data collection	None
Data analysis	RangeVision 3D studio 2022.1, ParaView 5.10.1

For manuscripts utilizing custom algorithms or software that are central to the research but not yet described in published literature, software must be made available to editors and reviewers. We strongly encourage code deposition in a community repository (e.g. GitHub). See the Nature Portfolio guidelines for submitting code & software for further information.

Data

Policy information about availability of data

All manuscripts must include a data availability statement. This statement should provide the following information, where applicable:

- Accession codes, unique identifiers, or web links for publicly available datasets
- A description of any restrictions on data availability
- For clinical datasets or third party data, please ensure that the statement adheres to our policy

All specimens are housed in accredited institutions and have unique identifying collection numbers (shown in the figure legends of Figs. 2 and 3), allowing them to be retrieved by other workers. All stls from optical scans of footprints shown in the manuscript figures have been uploaded to Figshare. The link to the folder containing all the files is https://doi.org/10.6084/m9.figshare.25869367. The Timetree database can be found at https://timetree.org

Research involving human participants, their data, or biological material

Policy information about studies with human participants or human data. See also policy information about sex, gender (identity/presentation), and sexual orientation and race, ethnicity and racism.

Reporting on sex and gender	n/a
Reporting on race, ethnicity, or other socially relevant groupings	n/a
Population characteristics	n/a
Recruitment	n/a
Ethics oversight	n/a

Note that full information on the approval of the study protocol must also be provided in the manuscript.

Field-specific reporting

Please select the one below that is the best fit for your research. If you are not sure, read the appropriate sections before making your selection.

☐ Life sciences ☐ Behavioural & social sciences ☒ Ecological, evolutionary & environmental sciences

For a reference copy of the document with all sections, see nature.com/documents/nr-reporting-summary-flat.pdf

Ecological, evolutionary & environmental sciences study design

All studies must disclose on these points even when the disclosure is negative.

Study description	Stone slabs carrying fossil footprints of early reptiles from the Carboniferous period were examined, the track-makers were identified as far as possible through comparison with previously described and named fossil footprints, and conclusions were drawn from these comparisons.
Research sample	One slab from Australia carrying two trackways and a single isolated footprint. This is the only known specimen from its locality and age. Additional material was described from a Polish museum collection.
Sampling strategy	On the Australian slab we sampled every footprint. In the Polish material, we picked out enough specimens (three were figured) to be able to make a secure taxonomic determination.
Data collection	Data were collected by means of photography and optical surface scanning. Both were performed by Grzegorz Niedzwiedzki.
Timing and spatial scale	n/a. The objects being studied (rocks with fossil footprints) are completely static and the timing of data collection has no effect on the study.
Data exclusions	No data exclusions
Reproducibility	The fossil specimens are all housed in accredited institutions and have unique accession numbers which are given in the paper. They can thus be retrieved and restudied by other researchers without difficulty.
Randomization	This is not relevant to the study as no statistical analyses requiring randomization were performed.
Blinding	This is not relevant to the study as no analyses requiring blinding were performed.

Did the study involve field work? ☒ Yes ☐ No

Field work, collection and transport

Field conditions	The fieldwork consisted of field-walking the banks of the Broken River near Barjarg, Victoria, Australia, looking for exposed bedrock blocks (sandstones of the Snowy Plains Formation) containing fossils.
Location	Banks of the Broken River near Barjarg, Victoria, Australia.
Access & import/export	Access was by permission of the landowner; Craig Eury and James Eason (the collectors) remain in touch with him and keep him

Access & import/export	informed about the fate of the slab. The slab has neither been imported nor exported, but remains in Victoria.
Disturbance	None. The slab was loose and exposed on the surface, it could simply be picked up.

Reporting for specific materials, systems and methods

We require information from authors about some types of materials, experimental systems and methods used in many studies. Here, indicate whether each material, system or method listed is relevant to your study. If you are not sure if a list item applies to your research, read the appropriate section before selecting a response.

Materials & experimental systems

n/a	Involved in the study
☒	☐ Antibodies
☒	☐ Eukaryotic cell lines
☐	☒ Palaeontology and archaeology
☒	☐ Animals and other organisms
☒	☐ Clinical data
☒	☐ Dual use research of concern
☒	☐ Plants

Methods

n/a	Involved in the study
☒	☐ ChIP-seq
☒	☐ Flow cytometry
☒	☐ MRI-based neuroimaging

Palaeontology and Archaeology

Specimen provenance	Permission was given verbally by the landowner. The specimen has not been exported or sold.
Specimen deposition	The specimen has been deposited with Museums Victoria in Melbourne, Victoria, Australia.
Dating methods	New dates were not obtained. The Snowy Plains Formation (part of the Mansfield Group) is dated by a combination of index fossils and regional tectonic history.

☐ Tick this box to confirm that the raw and calibrated dates are available in the paper or in Supplementary Information.

Ethics oversight	No ethical approval or guidance was sought or required, essentially because this was a chance discovery within a 'citizen science' context. However, as detailed in our separate ethics statement, close contact was kept with the Taungurung aboriginal community, the recognised Traditional Owners of the area where the slab was found, throughout the project. We ensured that use of Taungurung place names and crediting of the community in the paper complied with their wishes.

Note that full information on the approval of the study protocol must also be provided in the manuscript.

Plants

Seed stocks	*Report on the source of all seed stocks or other plant material used. If applicable, state the seed stock centre and catalogue number. If plant specimens were collected from the field, describe the collection location, date and sampling procedures.*
Novel plant genotypes	*Describe the methods by which all novel plant genotypes were produced. This includes those generated by transgenic approaches, gene editing, chemical/radiation-based mutagenesis and hybridization. For transgenic lines, describe the transformation method, the number of independent lines analyzed and the generation upon which experiments were performed. For gene-edited lines, describe the editor used, the endogenous sequence targeted for editing, the targeting guide RNA sequence (if applicable) and how the editor was applied.*
Authentication	*Describe any authentication procedures for each seed stock used or novel genotype generated. Describe any experiments used to assess the effect of a mutation and, where applicable, how potential secondary effects (e.g. second site T-DNA insertions, mosiacism, off-target gene editing) were examined.*
